# Complete genome sequence of ceftazidime-resistant *Burkholderia pseudomallei* strain 490f reveals a complex long repeat region

**DOI:** 10.1128/mra.00562-25

**Published:** 2025-08-11

**Authors:** Pacharapong Khrongsee, Kuttichantran Subramaniam, Ayalew Mergia, Apichai Tuanyok

**Affiliations:** 1 Department of Infectious Diseases and Immunology, College of Veterinary Medicine, University of Florida 3463, Gainesville, Florida, USA; 2 Emerging Pathogens Institute, University of Florida 145775, Gainesville, Florida, USA; 3 Faculty of Veterinary Science, Prince of Songkla University 26686, Hatyai, Songkhla, Thailand; The University of Arizona, Tucson, Arizona, USA

**Keywords:** melioidosis, *Burkholderia pseudomallei*, ceftazidime resistance

## Abstract

We report the complete genome of *Burkholderia pseudomallei* strain 490f, isolated from a ceftazidime treatment failure case in Northeast Thailand. Oxford Nanopore and Illumina sequencing techniques revealed two circular chromosomes. A 150 kb region on the small chromosome showed high coverage and complex repeats, which were manually resolved.

## ANNOUNCEMENT

This report describes the complete genome sequence of *Burkholderia pseudomallei* 490f, a ceftazidime-resistant isolate recovered in 1989 from a melioidosis patient in Northeast Thailand (1). The strain was originally isolated from the sputum culture of this patient 24 days after hospital admission. The strain was preserved in 20% glycerol at −80°C and shipped to the University of Florida in 2015 for genomic analysis. Upon receipt, the strain was sub-cultured on LB agar, and a single colony was selected and grown in LB broth at 37°C with shaking at 200 rpm for 18 h for genomic DNA extraction. Genomic DNA was extracted using the Wizard Genomic DNA Purification Kit (Promega) and sequenced using both Oxford Nanopore Technologies (ONT) and Illumina platforms. The ONT library was prepared using the PCR-free Ligation Sequencing Kit (SQK-NBD114.24) and NEBNext Companion Module (E7180L), and sequenced on a GridION system with an R10.4.1 flow cell. Guppy v6.5.7 (super-accurate mode) was used for base calling, demultiplexing, and adapter trimming, which generated 157,168 reads with an average length of 3,165 bp. Reads longer than 2,000 bp (69,268 reads; mean length 5,629 bp) were selected using SeqKit v2.4.0 (2), and used for *de novo* assembly with Canu v2.2 (3). Contigs were manually curated, and overlapping ends were trimmed in CLC Genomics Workbench v20.0.4, resulting in two complete circular chromosome scaffolds with an average ONT library coverage of 57.46×. Additionally, short-read sequencing was performed on an Illumina NextSeq 1000. Libraries were prepared using the NEBNext Ultra II FS DNA Library prep kit and Multiplex Oligos for Illumina (96 dual-indexed primers). Paired-end sequencing (2 × 300 bp) produced 18,124,202 reads. Quality control and adapter trimming were performed with BCL Convert v4.2.4. Reads were aligned to the ONT scaffolds using BWA v0.7.17 (4), and Pilon v1.23 was used to polish the genome, achieving a final Illumina coverage of 749× (5).

**Fig 1:**
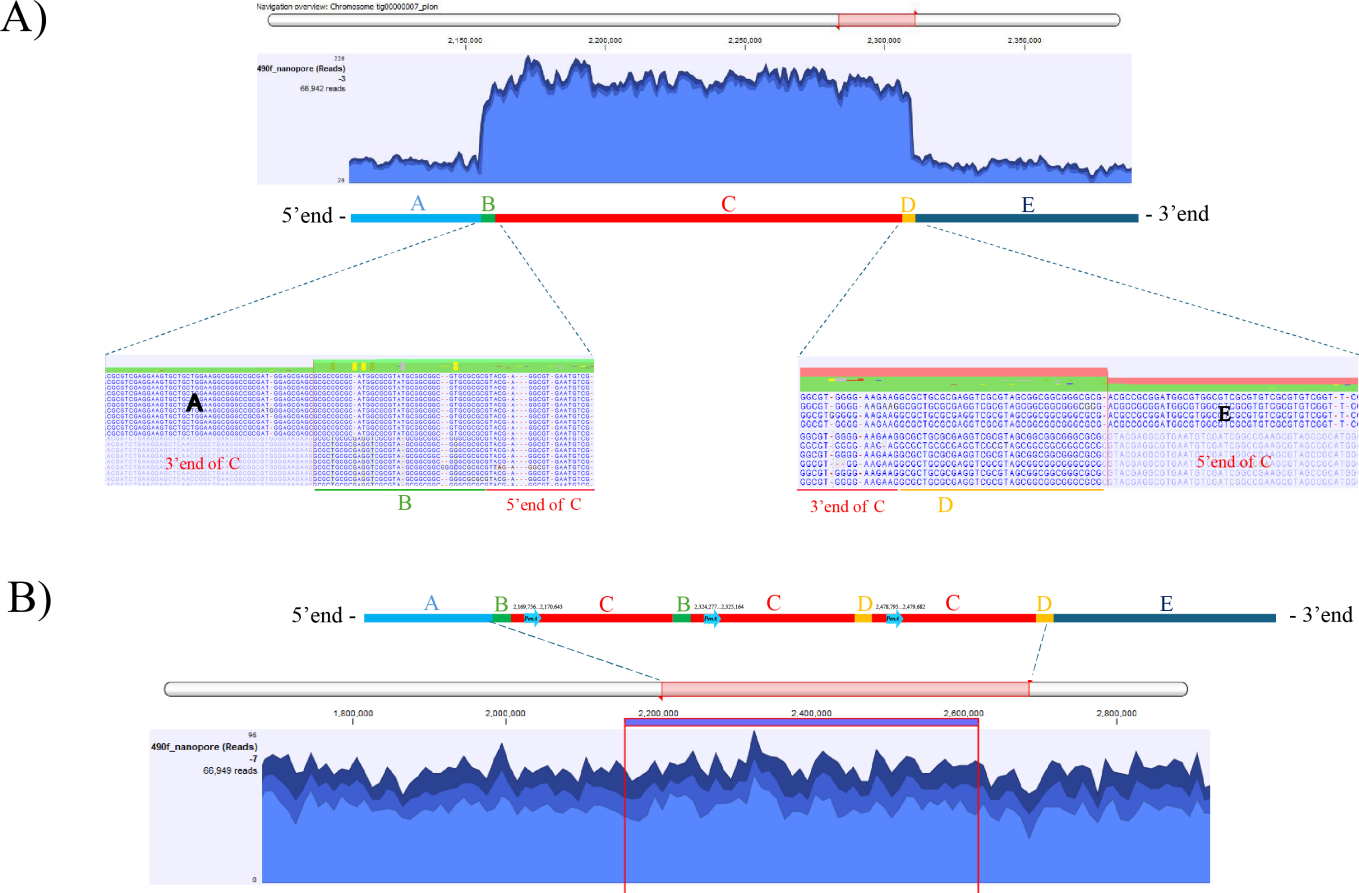
Repeat region and *penA* gene duplication in chromosome II of *B. pseudomallei* 490f: (**A**) elevated read depth observed across a repeat region, with mismatched alignments indicating repeat junctions; (**B**) corrected genome structure after manual curation of the repeat region, showing normalized read coverage. Three copies of *penA*, encoding a class A β-lactamase, were identified within this complex repetitive region.

While assessing genome completeness, an anomalously high ONT read depth (~3-fold higher than the genome-wide coverage) was observed across a ~150 kb region on chromosome II (coordinates 2.1–2.3 Mb). This region, comprizing segments B, C, and D (Fig. 1A), suggested the presence of complex segmental duplications (6). Notably, three copies of the *penA* gene, encoding a class A β-lactamase, were identified within the repetitive region. Visual inspection of long-read alignments revealed consistent mismatches at the flanking junctions: a portion of segment B aligned with Region A, while the remaining mismatched reads aligned to the 3′ end of segment C. Similarly, part of segment D aligned to Region E, with mismatches corresponding to the 5′ end of segment C, indicating a misassembly of the B–D region. To resolve this structure, long reads were used to reconstruct a corrected sequence. A consensus sequence was extracted and used to replace the misassembled region. After substitution, ONT reads were aligned, confirming normalized read coverage without structural anomalies (Fig. 1B). The revised genome was further polished using Illumina reads, as described above.

Genome annotation was performed using the NCBI Prokaryotic Genome Annotation Pipeline v6.9 (7). The final assembly consists of two circular chromosomes measuring 4,015,981 bp (G + C 67.85%) and 3,456,576 bp (G + C 68.67%), respectively. A total of 6,383 coding sequences, 63 tRNA, and 12 rRNA were identified. In a parallel study, we also found that ceftazidime resistance in this strain was linked to both *penA* gene duplication and a novel A172T amino acid substitution in PenA (8).

## Data Availability

The complete genome sequence of *Burkholderia pseudomallei* strain 490f was deposited at NCBI GenBank under the BioProject accession number PRJNA1258260, the Biosample accession number SAMN48311587, and the GenBank accession numbers CP190013 (chromosome I) and CP190014 (chromosome II). The raw reads were deposited in the Sequence Read Archive under the accession numbers SRR31877262 (Oxford Nanopore) and SRR32162008 (Illumina).
